# Focus on what works and why it works: bridging the gap between research knowledge and practical knowledge

**DOI:** 10.1007/s40037-017-0363-z

**Published:** 2017-06-07

**Authors:** Jan van Tartwijk, Manon Kluijtmans

**Affiliations:** 1 Department of Education, Faculty of Social and Behavioural Sciences, Utrecht University, Utrecht, The Netherlands; 2 Center for Education and Training, University Medical Center Utrecht, Utrecht, The Netherlands

In their contribution to Perspectives on Medical Education, Ng, Baker, and Leslie provide a novel and clear perspective on faculty development in health profession education (HPE) by conceptualizing it as a space for sharing research and practical knowledge [1]. The process of sharing these two types of knowledge implies that both are mobilized during faculty development and, in the end, become related. Successful faculty development then empowers faculty by enriching their contextualized experiential knowledge with research-based knowledge. According to Ng and colleagues, this enables them to become agents of positive change in their own system.

The key role that teacher involvement plays in the success of educational change has been widely recognized in the literature [2, 3]. The impact that research knowledge, i. e. evidence generated by systematic empirical research, can have as fuel for innovation of education has also been emphasized [4]. The million-dollar question that still needs to be answered concerns *how* to close the proverbial gap between research knowledge and practical knowledge [5] within the space professional development provides. Clues for its answer can be found in the literature on teacher professional development.

In an extensive review of the literature on the effectiveness of professional development in HPE, Steinert and her colleagues [6] identify a number of ‘key features’ that make professional development effective, including the role of experiential learning and feedback. They also emphasize the importance of working with peers. Similar reviews in the field of research on teachers and teaching [7, 8] found similar features, also emphasizing the importance of a focus on concrete issues related to instruction. Furthermore, models for teacher learning are available that provide suggestions for the utilization of research knowledge in teacher professional development [9, 10]. In all these models, reflection on application in practice is regarded as a key characteristic of effective professional development. Such reflection should not only focus on *what* works, but also on *why* it works, using the knowledge provided by previous research as a starting point.

Here, we want to advocate an approach for faculty development that aims to stimulate faculty to enrich their contextualized experiential knowledge with research-based knowledge, by stimulating them to not only focus on what works, but also on why it works.

Most faculty involved in medical education have been trained as clinicians and many of them also as researchers. Clinicians routinely exert evidence-based practice: integrating scientific evidence with clinical expertise and patient values. As researchers they are familiar with the routine of formulating research questions that are grounded in theory, systematically gathering data for analysis to answer these questions, and reflecting on their findings by considering how these findings match with existing knowledge based on research of others. All of these routines can be used in faculty development. When faculty design education they can use knowledge that is available in the research literature on (medical) education, they can systematically gather data on its success, and they can reflect on their evaluations together with their peers. Many of the key features of effective professional development are included in such approaches, and they could contribute to the development of what is referred to in the literature as scholarship of teaching and learning among faculty [11, 12]. In such a professional development space, research knowledge and practical knowledge would not only be mobilized, but would merge, laying the foundation for education in which research evidence is taken seriously [5].

Of additional importance, faculty might experience a coalescence of clinical, research and teaching roles by taking a scholarly approach to teaching, rather than experiencing teaching as an isolated (or conflicting) task. This could foster a positive narrative on teaching, which might strengthen teacher identity and motivation [13].
